# When measles is not benign: meningoencephalitis and status epilepticus in an unvaccinated adolescent (case report)

**DOI:** 10.11604/pamj.2026.53.96.50510

**Published:** 2026-02-23

**Authors:** Chaimae Bouhamdi, Zakia Douhi, Zouhayr Souirti, Echreiva Sidi El Moctar, Meryem Soughi, Sara Elloudi, Hanane Baybay, Fatima Zahra Mernissi

**Affiliations:** 1 Department of Dermatology, Hassan II University Hospital, Fez, Morocco,; 2 Department of Neurology, Hassan II University Hospital, Fez, Morocco

**Keywords:** Measles, meningoencephalitis, status epilepticus, viral exanthema, case report

## Abstract

Measles is a highly contagious viral exanthematous disease, caused by a Morbillivirus, that continues to cause outbreaks in under-immunized populations. While neurologic complications are rare, the progression to meningoencephalitis with convulsive status epilepticus is exceptional, particularly in immunocompetent adolescents. We report a 17-year-old unvaccinated male who presented with a febrile, descending asymptomatic maculopapular rash, lacking Koplik spots, followed by rapid onset of severe headache, photophobia, vomiting, and convulsive status epilepticus. Day-7 IgM was negative, and PCR was unavailable, prompting a broad viral, bacterial, and autoimmune workup, all of which were excluded. Cerebrospinal Fluid (CSF) showed mild protein elevation without pleocytosis, and repeat serology confirmed measles (IgM 1210 IU/mL; IgG 1980 IU/mL). The patient received meningeal-dose ceftriaxone, acyclovir, antiepileptics, and high-dose vitamin A per World Health Organization (WHO) recommendations, with complete neurologic recovery. This case highlights an uncommon but severe neurologic complication of measles in an unvaccinated adolescent, emphasizing the risk of diagnostic delay due to atypical features and early seronegativity. It reinforces the need for high clinical suspicion, broad etiologic evaluation, early empiric therapy, and sustained efforts to ensure complete vaccination.

## Introduction

Measles is a highly contagious systemic viral infection caused by a Morbillivirus, preventable through vaccination but nevertheless a persistent global threat, particularly as it becomes increasingly re-emergent in undervaccinated populations [1,2]. Beyond its classic prodrome and exanthem, measles can lead to a spectrum of rare but severe neurologic complications, including primary measles encephalitis, post-infectious encephalitis, measles inclusion body encephalitis (MIBE), and the late, progressive subacute sclerosing panencephalitis (SSPE) [2,4-9]. Severe neurologic involvement may manifest as meningoencephalitis with progression to convulsive status epilepticus, an exceptional cascade reported primarily in critically ill or immunocompromised patients and rarely in immunocompetent adolescents [4,5,7]. Early diagnosis is further challenged by atypical presentations, absence of Koplik spots, and the early false-negative IgM serology, especially when PCR testing is unavailable in resource-limited settings [3,7]. We report a non-vaccinated adolescent with measles presenting atypically, evolving into fulminant neurologic disease. This highlights diagnostic complexity and reinforces the critical importance of vaccination and clinical vigilance, especially amid fluctuating vaccine coverage and rising risk of severe neurologic outcomes such as SSPE in unvaccinated individuals [1,8,9].

## Patient and observation

**Patient information:** a 17-year-old male with no prior history presented with febrile maculopapular rash and convulsive status epilepticus, raising suspicion for measles-associated meningoencephalitis. His mother reported, verbally, adherence to routine vaccination per National Immunization Program (NIP), but records were unavailable, and verification was therefore planned with health authorities. Initial symptoms were flu-like: fatigue, myalgias, low-grade fever, and left otalgia without sore throat, dysuria, or cough. Three days later, he developed an asymptomatic descending febrile exanthem. After another three days, intense diffuse refractory headaches, photophobia, and projectile vomiting emerged. Within 48 hours, a generalized seizure without regained consciousness declared convulsive status epilepticus and meningoencephalitis. He was sedated and intubated in ICU. At day 7 from rash onset, measles serology was IgM-negative. Contrast-enhanced cranial CT was normal. Cerebrospinal fluid (CSF) was clear, with a low white cell count (9 cells/mm^3^), negative cultures and multiplex PCR, and mildly elevated protein (0.69 g/L) and glycorrhachia (0.79 g/L) with normal blood glucose (4.5 mmol/L), and normal chloride levels (120 mmol/L). Due to uncertainty between viral and bacterial meningoencephalitis, empiric therapy included acyclovir (20 mg/kg every 8 hours), ceftriaxone (3 g every 12 hours), sodium valproate (500 mg TID), clobazam (5 mg TID for 7 days, then tapered over 4 weeks), and high-dose vitamin A (200,000 IU on Days 1, 2, and 30). Thoracic CT revealed bilateral posterobasal consolidations suggestive of aspiration pneumonia. Cultures from bronchial aspirate and urine were negative; ENT swabs showed only saprophytic flora. After seven days, the patient regained consciousness and was transferred to our dermatology department.

**Figure 1:**
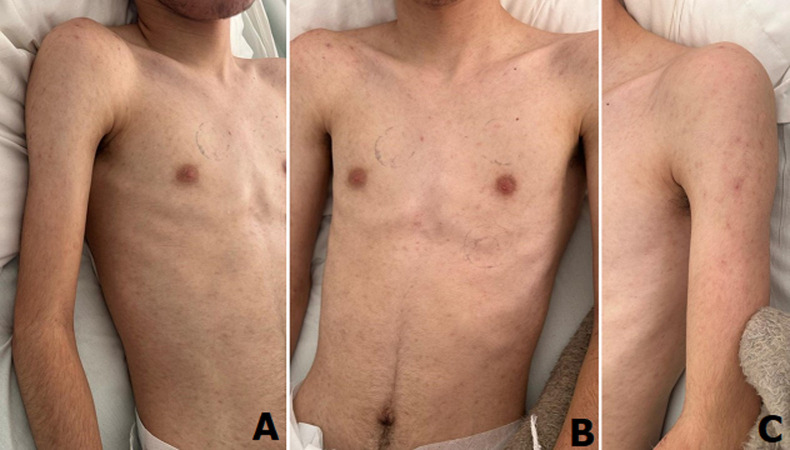
A, B, C) frontal view of the trunk and upper limbs showing a roseoliform maculopapular exanthema composed of non-pruritic erythematous macules and papules

**Figure 2:**
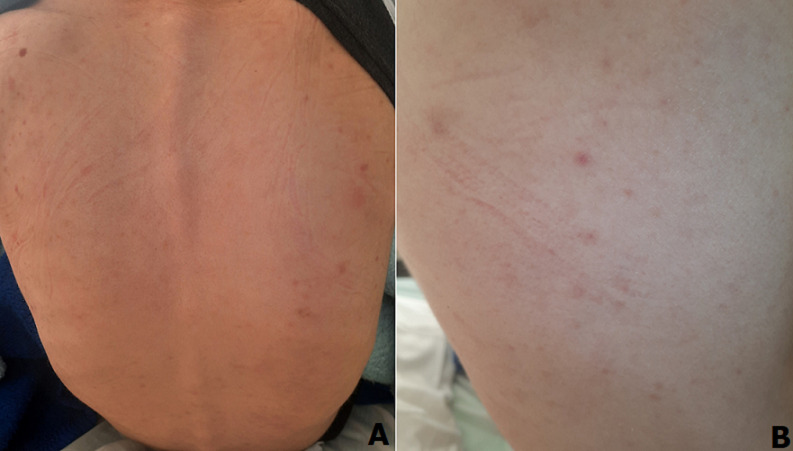
A) posterior and B) lateral views of the trunk showing the same roseoliform maculopapular exanthema, with diffuse non-pruritic erythematous macules and papules

**Figure 3:**
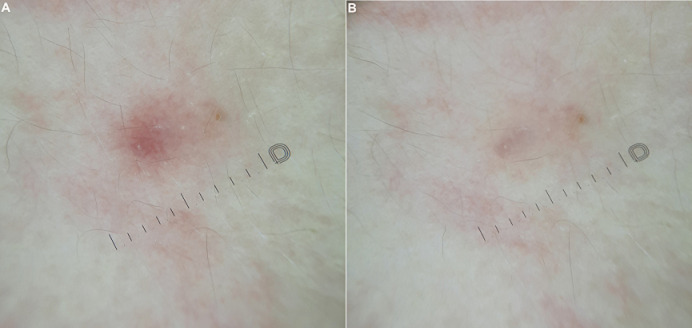
polarized contact dermoscopy of an erythematous maculopapular lesion; A) before vitropression, showing blanchable erythema; B) after gentle vitropression, confirming the non-purpuric nature of the lesion

**Figure 4:**
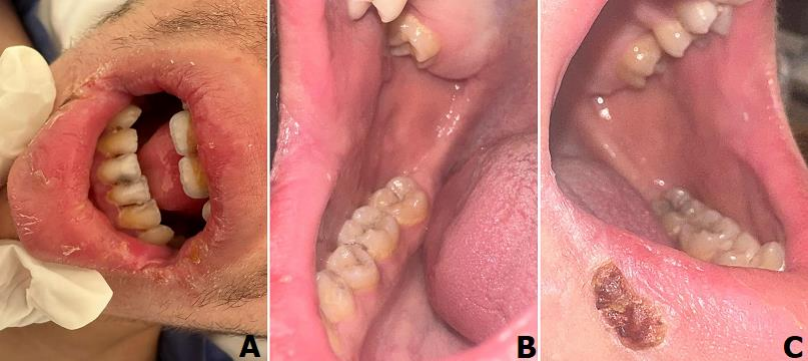
A, B, C) oral examination showing absence of Koplik spots; perioral ulceration and cheilitis were attributed to intubation

**Clinical findings:** on admission, he was alert (GCS 15), afebrile (36°C), hypotensive (89/57 mmHg), asthenic, with stable vitals and no further seizures. Neurologic examination revealed only slight right-sided dysmetria on finger-to-nose testing. Dermatologic exam revealed a Fitzpatrick III phototype with roseola-like maculopapular rash on trunk and upper limbs and residual hyperpigmentation (Figure 1, Figure 2). Dermoscopy showed erythematous, non-purpuric lesions (Figure 3). Perioral ulcer and cheilitis were attributed to intubation. No Koplik spots (Figure 4), or lymphadenopathy. Conjunctival hyperemia was managed symptomatically.

**Diagnostic assessment:** despite clinical suspicion, lack of local cases and negative early serology prompted a broad differential. Toxidermias (e.g., PEAG) were excluded due to absent pustules. DRESS was ruled out by lack of drug exposure, periorbital edema, polymorphic rash, or systemic involvement. Viral etiologies including measles, HHV-6/7 were explored. Although the patient had recurrent herpes labialis, HHV-1 reactivation was unlikely in the absence of vesicular lesions. HIV was ruled out based on negative serology and undetectable viral load. EBV was unlikely with no pharyngitis, lymphadenopathy, or hepatosplenomegaly; IgG was negative. CMV serology showed positive IgG and negative IgM, indicating past infection. Hepatitis and syphilis were excluded clinically and serologically. Bacterial etiologies were ruled out: meningococcemia (no purpura or neck stiffness), rickettsial disease (no eschar or acral signs), and typhoid fever, tuberculosis, and endocarditis (all three due to absence of exposure, systemic signs, and negative cultures). Autoimmune causes, including systemic lupus erythematosus and Behçet´s disease, were excluded based on absence of systemic symptoms and defining signs like aphthosis and pseudofolliculitis, negative immunologic workup (ANA, anti-dsDNA, ENA, ANCA, pathergy test) and a normal MRI. Paraneoplastic etiologies were unsupported by clinical context.

Laboratory tests revealed stable hemoglobin, normal leukocyte and neutrophil counts, and lymphopenia (600-1300 cells/mm^3^). CRP ranged from 54 to 133 mg/L. CPK was transiently elevated to three times normal (620 U/L), with CPK-MB peaking at eight times normal (191 U/L). ECG and cardiac biomarkers were normal, and the elevated CK-MB was attributed to skeletal muscle injury secondary to convulsive status epilepticus. Procalcitonin was mildly elevated (0.36 ng/mL). Renal and hepatic function, ESR, beta-2 microglobulin, immunoglobulin levels, EEG, brain MRI and thoraco-abdomino-pelvic CT were all normal.

**Figure 5:**
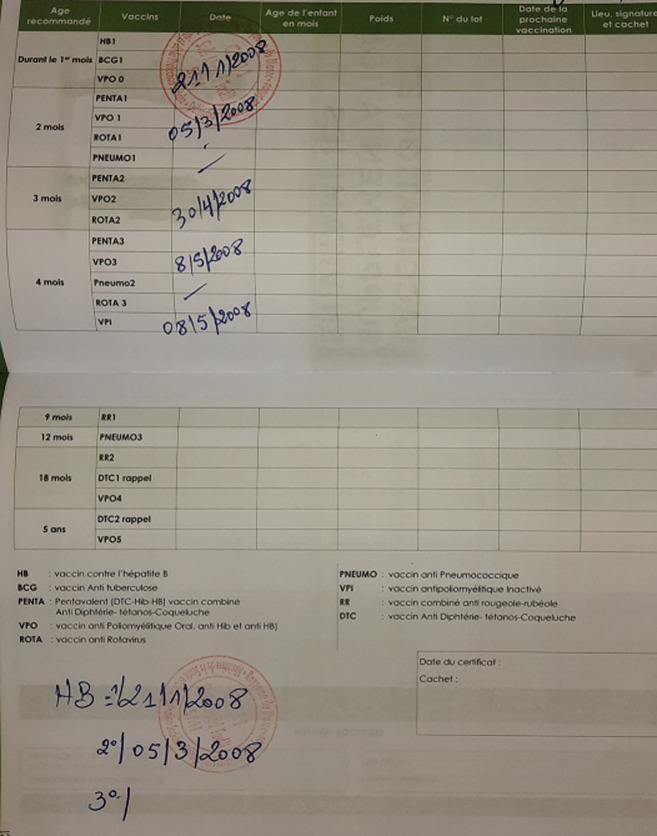
retrieved vaccination records confirming that both measles-containing vaccine (MMR) doses scheduled at 9 and 18 months were missed

**Therapeutic interventions:** the patient remained in isolation under empiric therapy. Epidemiologic authorities were notified to repeat measles serologic testing. Retrieved vaccination records confirmed only early infant immunizations, with both measles-containing vaccine MMR doses at 9 and 18 months missed (Figure 5).

**Diagnosis:** repeat serology at day 21 from rash onset revealed markedly elevated measles IgM (1210 IU/mL) and IgG (1980 IU/mL), consistent with seroconversion after the initial IgM-negative serologic assay at day 7. This established the diagnosis of acute measles in an unvaccinated adolescent, complicated by meningoencephalitis with progression to convulsive status epilepticus. Measles-specific RT-PCR on respiratory or urine specimens was not attainable when indicated despite escalation, thereby precluding early molecular confirmation.

**Follow-up and outcome of interventions:** recovery was complete, with resolution of the eruption, which evolved with fine desquamation and residual hyperpigmented macules. The patient was discharged on valproate and referred for neurology follow-up.

**Patient perspective:** after regaining consciousness and being informed of the diagnosis, the patient expressed relief at understanding the cause of his severe neurologic episode. He acknowledged the seriousness of the illness and agreed to adhere to long-term neurologic surveillance given the potential delayed complications of measles.

**Informed consent:** written informed consent for publication of this case and accompanying clinical and dermoscopic images was obtained from the patient´s father. The patient, although a minor, provided verbal and written assent.

## Discussion

Measles is an acute, highly contagious systemic viral infection caused by a Morbillivirus of the Paramyxoviridae family [2]. Transmission occurs via airborne droplets and leads to a systemic viremia capable of affecting multiple organs, including the central nervous system (CNS) [2,7]. Although classically considered a childhood exanthematous illness, measles is far from benign and continues to pose a major global concern, particularly in regions of low vaccination coverage, where recent epidemiologic data show recurrent outbreaks and increased risk of severe complications [1]. In its classic form, measles begins with a prodrome of fever, cough, coryza and conjunctivitis, followed by Koplik spots and a descending erythematous maculopapular exanthem [7]. Neurologic involvement represents the rare but most severe and feared end of the measles disease spectrum, ranging from acute primary measles encephalitis to post-infectious encephalitis, measles inclusion body encephalitis (MIBE) and the late, progressive subacute sclerosing panencephalitis (SSPE), a fatal neurodegenerative condition occurring years after infection [2,4,5,9]. Primary measles encephalitis occurs in approximately one per thousand cases and typically presents within days of the exanthem with fever, headache, altered consciousness or seizures [3,9]. Aggressive forms may progress to meningoencephalitis and, in exceptional cases, to convulsive status epilepticus, a cascade described mainly in critically ill or immunocompromised individuals and only rarely in immunocompetent adolescents [4,5,7].

In addition to the atypical clinical features, early serology further complicated diagnosis. Day 7 measles IgM was negative, a documented limitation of early testing or delayed seroconversion described in primary measles encephalitis and severe forms [3,8], particularly in resource-limited settings where PCR is unavailable and diagnosis relies on repeated serologic testing [3,7]. Given the neurologic severity, a broad differential diagnosis was necessary. Acute toxidermias were ruled out, including PEAG in the absence of pustules and DRESS in the absence of drug exposure, periorbital edema, polymorphic rash or systemic involvement. HHV-6 and HHV-7 infections were initially considered for their neurotropism and roseoliform exanthem, but the rash morphology and absence of systemic viral features rendered this unlikely [10]. Herpes simplex reactivation was improbable in the absence of vesicular lesions. EBV, CMV, HIV, hepatitis and syphilis were excluded serologically, with CMV IgG positivity reflecting past infection only. Autoimmune etiologies, including systemic lupus erythematosus and Behçet´s disease, were excluded by the absence of systemic features and a negative immunologic workup (ANA, anti-dsDNA, ENA, ANCA) together with a negative pathergy test. The combination of clinical features, neurologic evolution and delayed seroconversion oriented strongly toward measles meningoencephalitis [4-7].

Neuroimaging was unremarkable, a finding frequently reported in primary measles encephalitis [7,9]. Cerebrospinal fluid analysis revealed mildly elevated protein with normal cell count and sterile cultures, a pattern compatible with viral meningoencephalitis and previously described in acute measles central nervous system involvement [6,7,9]. Early serology remained negative, consistent with documented false-negative IgM in the first week of infection [3,7]. PCR, the preferred early diagnostic modality, was not available locally, making repeat serology essential for confirmation [3,7]. Dermoscopy revealed non-purpuric erythematous lesions, helping exclude a vasculitic process, although dermoscopy is not a standard diagnostic tool in measles. Repeat serologic testing showed markedly elevated IgM and IgG, establishing the diagnosis of measles meningoencephalitis progressing to status epilepticus [7]. Diagnostic confirmation relies on the convergence of a compatible clinical presentation, evidence of seroconversion or rising IgM and IgG titers, PCR when available, and systematic exclusion of alternative infectious or autoimmune etiologies, as described in published series of measles encephalitis and meningoencephalitis [4-7]. In primary measles encephalitis, cerebrospinal fluid is frequently normal or shows only mild protein elevation, as observed in our patient [4,6,7].

In this case, the clinico-biological constellation, including a descending maculopapular rash, early neurologic symptoms (headache, photophobia, vomiting), normal neuroimaging, a characteristic CSF profile, and delayed serologic positivity, formed a coherent picture consistent with primary measles meningoencephalitis [4-7]. The absence of Koplik spots and the atypical exanthem significantly contributed to diagnostic delay [7]. Negative serologic and immunologic workup for HHV-6, HHV-7, EBV, HIV, hepatitis, syphilis, systemic lupus erythematosus and Behçet´s disease, together with an unremarkable brain MRI, supported exclusion of competing diagnoses. The temporal association between rash onset and progression to convulsive status epilepticus further strengthened the suspicion of acute measles neuroinvasion [4,7].

Public health-wise, this case highlights immunization gaps. Although vaccination was assumed, both MMR doses at 9 and 18 months had been missed. Completion of the recommended two-dose MMR schedule is essential to ensure durable protective immunity [1]. This gap left the patient vulnerable to severe disease and possible late sequelae such as SSPE [8,9]. Despite full recovery, SSPE remains a serious concern. This progressive neurodegenerative disorder, caused by persistent measles virus in the CNS, may develop years later, particularly in unvaccinated individuals [8,9]. Though more frequent in children under two, adolescent cases are reported [8]. Long-term neurologic surveillance and patient education are therefore essential.

Treatment relied on recommended practice for suspected viral meningoencephalitis with status epilepticus. Empiric meningeal-dose acyclovir and ceftriaxone were initiated due to diagnostic uncertainty and in accordance with standard care in acute meningoencephalitis [4,7]. Sodium valproate and clobazam were administered in line with neurologic emergency protocols for convulsive status epilepticus. High-dose vitamin A was administered according to WHO recommendations for severe measles, given its documented benefit in reducing morbidity and improving immune recovery [3]. Isolation, supportive care and early notification of public health authorities were essential given his unvaccinated status. This comprehensive approach likely contributed to the favorable neurologic outcome.

## Conclusion

This case demonstrates the potential for measles to trigger a fulminant, life-threatening neurologic cascade in an immunocompetent unvaccinated adolescent, even in the absence of classic features. This atypical constellation exposes major diagnostic pitfalls and underscores the need for clinicians to maintain heightened suspicion in similar presentations, particularly amid declining vaccine coverage. It further emphasizes the importance of repeated serologic testing, prompt empiric management, and strict completion of measles immunization schedules.

## References

[1] Parums DV (2024). A review of the resurgence of measles, a vaccine-preventable disease, as current concerns contrast with past hopes for measles elimination. Med Sci Monit.

[2] Watanabe S, Shirogane Y, Sato Y, Hashiguchi T, Yanagi Y (2019). New insights into measles virus brain infections. Trends Microbiol.

[3] Diwan MN, Samad S, Mushtaq R, Aamir A, Allahuddin Z, Ullah I (2022). Measles induced encephalitis: recent interventions to overcome the obstacles encountered in the management amidst the COVID-19 pandemic. Diseases.

[4] Rafat C, Klouche K, Ricard JD, Messika J, Roch A, Machado S (2013). Severe measles infection: the spectrum of disease in 36 critically ill adult patients. Medicine (Baltimore).

[5] Garg RK, Mahadevan A, Malhotra HS, Rizvi I, Kumar N, Uniyal R (2019). Subacute sclerosing panencephalitis. Rev Med Virol.

[6] El-Far F, Sztajnbok J, Marotto PCF, Seguro AC (2000). Meningoencefalite na fase aguda do sarampo: relato de seis casos [Meningoencephalitis in the acute phase of measles. Report of 6 cases]. Arq Neuropsiquiatr.

[7] Al-Qayoudhi A, Al-Kindi H, Meki N, Al-Maani A (2016). Acute measles encephalitis in an immigrant Syrian child: case report and review of the literature. Oman Med J.

[8] Khetsuriani N, Sanadze K, Abuladze M, Tatishvili N (2020). High risk of subacute sclerosing panencephalitis following measles outbreaks in Georgia. Clin Microbiol Infect.

[9] Ferren M, Horvat B, Mathieu C (2019). Measles encephalitis: towards new therapeutics. Viruses.

[10] Agut H, Bonnafous P, Gautheret-Dejean A (2016). Human herpesviruses 6A, 6B, and 7. Microbiol Spectr.

